# Interplay between stress and cancer—A focus on inflammation

**DOI:** 10.3389/fphys.2023.1119095

**Published:** 2023-03-20

**Authors:** Sanja Vignjević Petrinović, Maja S. Milošević, Dragana Marković, Sanja Momčilović

**Affiliations:** ^1^ Group for Neuroendocrinology, Institute for Medical Research, National Institute of Republic of Serbia, University of Belgrade, Belgrade, Serbia; ^2^ Group for Immunology, Institute for Medical Research, National Institute of Republic of Serbia, University of Belgrade, Belgrade, Serbia

**Keywords:** chronic stress, cancer, inflammation, proinflammatory cytokines, microenvironment

## Abstract

Stress is an integral part of life. While acute responses to stress are generally regarded as beneficial in dealing with immediate threats, chronic exposure to threatening stimuli exerts deleterious effects and can be either a contributing or an aggravating factor for many chronic diseases including cancer. Chronic psychological stress has been identified as a significant factor contributing to the development and progression of cancer, but the mechanisms that link chronic stress to cancer remain incompletely understood. Psychological stressors initiate multiple physiological responses that result in the activation of the hypothalamic-pituitary-adrenal (HPA) axis, sympathetic nervous system, and the subsequent changes in immune function. Chronic stress exposure disrupts the homeostatic communication between the neuroendocrine and immune systems, shifting immune signaling toward a proinflammatory state. Stress-induced chronic low-grade inflammation and a decline in immune surveillance are both implicated in cancer development and progression. Conversely, tumor-induced inflammatory cytokines, apart from driving a tumor-supportive inflammatory microenvironment, can also exert their biological actions distantly *via* circulation and therefore adversely affect the stress response. In this minireview, we summarize the current findings on the relationship between stress and cancer, focusing on the role of inflammation in stress-induced neuroendocrine-immune crosstalk. We also discuss the underlying mechanisms and their potential for cancer treatment and prevention.

## Introduction

Stress is an inevitable component of daily life that can affect health in a variety of ways. In order to survive, any individual is required to adequately respond and adapt to an everchanging environment. Physical or psychological stimuli that disrupt homeostasis are known as stressors. The exposure to stressors results in complex interactions among nervous, endocrine, and immune systems, regarded as a stress response. The initial response to a stressful stimulus is adaptive and encompasses the activation of the sympathetic-adreno-medullar (SAM) system, the hypothalamic-pituitary-adrenal (HPA) axis, and the immune system (Godoy et al., 2018). Hence, in response to real or perceived threats to homeostasis, the information about the stressor is processed by an intricate network of brain circuitry that includes the hippocampus, amygdala, and prefrontal cortex (McEwen and Gianaros, 2010), subsequently initiating the physiological mechanisms of adaptation mediated mainly by catecholamines and glucocorticoids. Adrenal medullary hormones—epinephrine as well as norepinephrine, which is also synthetized in the locus coeruleus and released by sympathetic nerve terminals, orchestrate the ‘fight or flight’ response during stress. In addition to the autonomic nervous system, HPA axis acts as the key player of stress response *via* the regulation of glucocorticoid secretion (Godoy et al., 2018). This HPA-mediated stress response is characterized by hypothalamic release of corticotropin-releasing hormone (CRH) that acts on the anterior pituitary to stimulate the secretion of adrenocorticotropic hormone (ACTH), which in turn leads to the adrenal secretion of cortisol (human) or corticosterone (rodents). Synergically, the activation of HPA axis and SAM system results in increased breakdown of glucose and promotes redistribution of energy in a wide range of tissues/organs, including the brain (Godoy et al., 2018). New data reveals that distinct brain regions drive the specific leukocyte trafficking pattern during acute psychological stress, therefore modulating the immune function (Poller et al., 2022). This stress-induced modulation of immune function is highly dependent on stressor intensity and duration (Dhabhar, 2014). In addition, stress experienced prior to novel antigen exposure or during early stages of immune activation results in a significant immunoenhancement, while immunosuppression may be observed at late stages of an immune response (Dhabhar, 2008).

Unlike acute stress response, prolonged or repeated exposure to stressors may lead to a maladaptive response and can be detrimental to health. However, it is important to emphasize that stress responses in an individual is likely to be a continuum ranging from potentially adaptive to predominantly maladaptive state, deeply affected by the intensity, duration, predictability, and controllability of the stressor (Suri and Vaidya, 2015). Apart from these factors related to the stressor itself, the factors intrinsic to the individuals, such as age, sex and genetic background, significantly shape the stress response (Novais et al., 2016). Thus, psychological stressors induce robust increases in circulating inflammatory factors and emerging evidence suggests that sex differences in stress-induced inflammatory response may have therapeutic implications (Martinez-Muniz and Wood, 2020). Furthermore, short-term exposure to psychological stressors mainly enhances the immune response, whereas repeated/chronic exposure results in suppression of immune function (Dhabhar, 2014). Acute stress response induces a rapid redistribution of immune cells among different body compartments, which is similar across species, suggesting an evolutionarily conserved mechanism that confer an adaptive advantage (Dhabhar, 2018). Stress hormones norepinephrine and epinephrine mobilize immune cells from the bone marrow, spleen, lung, lymph nodes into the bloodstream soon after the beginning of stress (within 30 min). Later during stress (30 min to a few hours), glucocorticoids and epinephrine drive immune cell migration out of the blood to target tissues such as the skin, secondary lymphoid tissues, sites of ongoing or *de novo* immune activation, thus significantly enhancing the speed and efficacy of the immune response. Functional implications of stress-induced immune cells redistribution are reflected in significantly enhanced immune responses within target tissues which are enriched with leukocytes during stress. It is important to note that leukocytes exhibit distinct sensitivities and redistribution kinetics in response to each stress hormone, depending on the type of cell and its functional characteristics (Dhabhar et al., 2012). In addition, the magnitude of stress-induced immune cell redistribution is largely dependent on stressor characteristics including its duration. In contrast to acute stress-elicited immunoenhancement in target tissues/compartments, chronic stress generally suppresses and/or dysregulates immune responses (Dhabhar, 2008). Exposure to chronic stress commonly leads to a decrease in immune cell numbers and function, a significant immunosuppression, as well as to an altered type 1/type 2 cytokine balance. Stress-induced immune dysfunction contributes to sustained low-grade inflammation that is closely associated with common chronic diseases including cancer. On the other hand, cancer itself leads to an inflammatory milieu with systemic manifestations (Demers et al., 2018), thus altering HPA axis activation and appropriate stress-induced cortisol secretion (Nolten et al., 1993). In this minireview we will discuss a mutual crosstalk between stress and cancer, focusing on the role of inflammation as a potential therapeutic target in stress/cancer relationship.

## Chronic stress and cancer—A bidirectional relationship

Epidemiological and clinical studies have provided strong evidence for links between chronic stress and increased risk of cancer incidence and mortality (Moore et al., 2022). On the other hand, coping with cancer and going through intensive anti-cancer treatment is a significant cause of chronic stress for cancer patients. Under chronic stress conditions, a region-specific neuronal remodeling has been demonstrated in multiple brain regions including the hippocampus, amygdala, and prefrontal cortex (McEwen et al., 2016). Thus, repeated stress exposure induces sustained synaptic plasticity in the prefrontal cortex (Colyn et al., 2019) and elicits contrasting patterns of dendritic alterations in the amygdala and hippocampal neurons (Patel et al., 2018). Furthermore, repeated exposure to the most stressors increase hypothalamic CRH gene and protein expression, enhance cellular excitability by increasing the density of catecholaminergic and glutamatergic terminals on CRH neurons, thus leading to chronic activation of HPA axis with subsequent glucocorticoid hypersecretion and sensitized stress responses (Herman and Tasker, 2016). In addition to prolonged HPA axis activation, chronic exposure to stressors causes the alterations in locus coeruleus-norepinephrine function and growing evidence suggests that the enhanced sympathetic activity also contributes to glucocorticoid hypersecretion following chronic stress (Lowrance et al., 2016).

**FIGURE 1:**
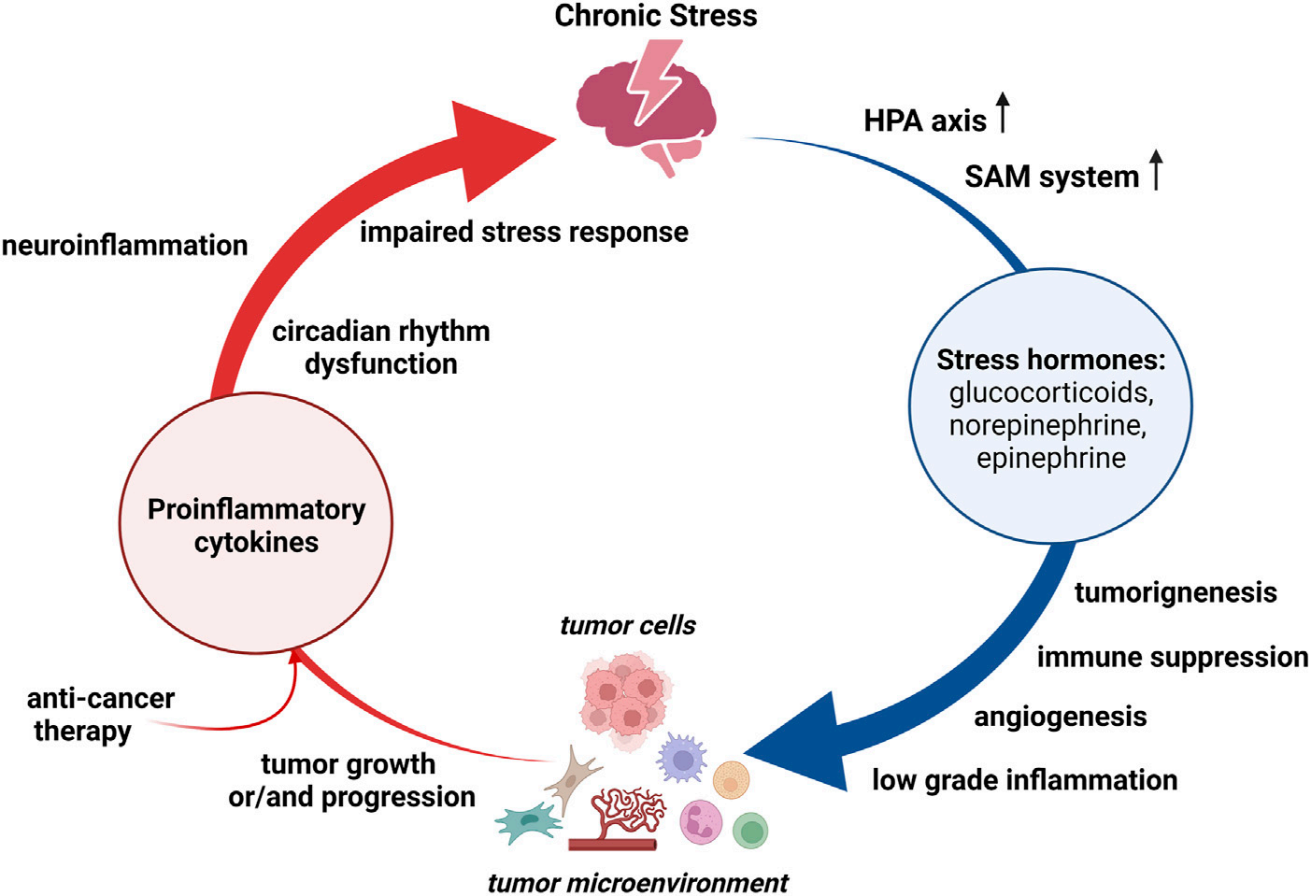
Bidirectional relationship between chronic stress and cancer. Chronic stress persistently activates hypothalamus-pituitary-adrenal (**HPA**) axis and sympathetic-adreno-medullar (**SAM**) system. Stress hormones can promote tumorigenesis, suport cancer growth and/or progression, and regulate tumor microinvironment. Both, chronic stress and tumor, induce production of proinflammatory cytokines, which may cause neuroinflammation and thereby altering stress responses. Anti-cancer therapy may also contribute to a persistant proinflammatory state.

Chronic stress can promote cancer development, progression and therapy resistance *via* multiple mediators and underlying mechanisms, such as continuous release of stress hormones, immune suppression, and persistent low-grade inflammation (Dai et al., 2020). Both HPA and SAM systems are persistently activated during chronic stress, resulting in high levels of stress hormones in different tissues including the solid tumors (Tian et al., 2021). Stress hormones may promote tumorigenesis by inducing DNA damage and suppressing protein p53 function, and support cancer growth and/or progression directly or indirectly by influencing the tumor microenvironment (Figure 1). Thus, treatment of human oral epithelial cells with either norepinephrine or cortisol leads to single-strand breaks and alkali-label side breaks in the DNA, and this DNA damage is prevented by pre-treatment with beta-adrenergic receptor antagonist propranolol and glucocorticoid receptor antagonist RU486, respectively (Valente et al., 2021). Furthermore, Feng et al. (2012) have demonstrated that increased glucocorticoid concentrations during chronic restraint stress reduced p53 levels and function, which in turn contributed to irradiation-induced tumorigenesis. Most recently, Zong et al. (2022) have pointed toward a key role of beta-adrenergic signaling in chronic stress-induced malignant transformation of gastric epithelial cells through the induction of p53 protein degradation. Aside from the impact on tumorigenesis, stress hormones can stimulate the proliferation of cancer cells by activating various signaling pathways and promoting cell division. Experimental data have revealed that catecholamines stimulate the proliferation of colorectal carcinoma cells *in vitro* and promote tumor growth *in vivo via* activation of extracellular signal-regulated kinases-1/2 (ERK1/2) by adrenergic signaling (Lin et al., 2013). Likewise, treatment with beta-adrenergic receptor antagonist propranolol inhibits ERK1/2 pathway and induces G1/S phase cell cycle arrest and apoptosis in gastric cancer cells, confirming the prominent role of catecholamines in cancer cell proliferation (Koh et al., 2021). The significant role of adrenergic signaling has also been recognized in the proliferation of breast cancer cells (Obeid and Conzen, 2013). Nevertheless, the inhibitory effect of the beta-adrenergic receptor agonist on breast cancer cells has also been reported, suggesting that the effects of catecholamines on cancer cell growth may vary depending on specific tumor type, receptor expression and selectivity of beta-adrenergic receptor agonists and antagonists (Yap et al., 2018). Glucocorticoids also increase proliferation in different cancer cell lines by activating the AKT and mitogen-activated protein kinase (MAPK) pathways (Gündisch et al., 2012). In addition to these direct effects on malignant cells, stress hormones may also regulate tumor microenvironment and consequently support *de novo* angiogenesis, tumor growth and aggressiveness (Calvani et al., 2015; Quatrini et al., 2021; Tian et al., 2021). For example, the activation of beta-adrenergic receptors in melanoma cells enhances their response to stromal fibroblasts and macrophages, increases cell motility and induce stem-like properties. Moreover, beta-adrenergic signaling in melanoma neighboring cells initiates stromal reactivity, resulting in *de novo* angiogenesis and sustained tumor growth (Calvani et al., 2015). In accordance, the increased stromal expression of matrix metalloproteases and vascular endothelial growth factor A, mediated by beta-adrenergic signaling, can promote tumor invasion and metastasis *via* extracellular matrix degradation and angiogenesis (Azevedo Martins et al., 2020; Tian et al., 2021). Furthermore, adrenergic receptors activation stimulates the growth and proliferation of cancer-associated fibroblasts and subsequently increases the concentration of growth factors in tumor microenvironment (Tian et al., 2021). Among the multiple cell types within tumor microenvironment the immune cells are recognized as the leading players that possess both pro- and anti-tumor activities. Chronic stress considerably affects hematopoietic stem/progenitor cells (Heidt et al., 2014; Vignjević Petrinović et al., 2020; Barrett et al., 2021) and mature immune cells, altering the hematopoietic homeostasis and dysregulating both innate and adaptive immune responses (Dhabhar, 2014). In general, different populations of hematopoietic cells within tumor microenvironment such as: myeloid-derived suppressor cells, tumor-associated macrophages (M2 polarized), polymorphonuclear cells, type 2 and 3 of innate lymphoid cells, mast cells, regulatory T cells, and nucleated erythroid cells are considered to exert the immunosuppressive effects, whereas tumor-infiltrating lymphocytes, dendritic cells and natural killer cells are associated with an anti-tumor activity (Salemme et al., 2021; Vignjević Petrinović et al., 2022). Thus, chronic stress can promote cancer progression by accumulating the immune cells that exert immunosuppressive effects (An et al., 2021) as well as suppressing the number and function of immunoprotective cells (Dhabhar, 2014). Furthermore, chronic stress induces sustained production of proinflammatory cytokines, such as: interleukin (IL)-6, IL-1β, or tumor necrosis factor (TNF)α, and emerging evidence suggests that prolonged exposure to psychological stress triggers the reprogramming of myeloid cells towards a hyperinflammatory state (Barrett et al., 2021).

Inflammation is now considered as a hallmark of cancer. A role of inflammation has been widely studied in many aspects of cancer development including initiation, promotion, and progression, and both positive and negative correlation between inflammation and cancer have been reported (Liu et al., 2021), Conversely, cancer itself alters the balance of cytokine production toward an inflammatory milieu (Burkholder et al., 2014). Apart from cancer itself, a persistent proinflammatory state in cancer patients is often the result of either targeted therapy or palliative treatment (Ahmad et al., 2021; Francesco et al., 2021). Regardless of their origin, the pro-inflammatory cytokines produced in the periphery can enter the circulation, permeate the blood-brain barrier, increase the production of local inflammatory mediators, and subsequently impair stress responses (Figure 1). Thus, cancer may cause neuroinflammation and affect stress responses by altering different aspects of neural function, such as circadian rhythm dysfunction, sleep disturbances, aberrant glucocorticoid production, and dysregulation of neural network activity (Pyter, 2016). In accordance, McCaffrey et al. (2022) have most recently demonstrated that, analogously to chronic stress, cancer itself activates microglia through stress neurocircuitry.

Clinical data strongly indicate a dysregulated HPA axis function and abnormal secretion of cortisol in cancer patients (Weinrib et al., 2010). In these patients, cortisol levels can be altered due to several factors including the cancer itself, treatments such as chemotherapy, and psychological stress associated with a cancer diagnosis (Francesco et al., 2021). Thus, an altered cortisol diurnal rhythm has been demonstrated in patients with cancer prior to surgery (Schrepf et al., 2015; Chang and Lin, 2017), and an aberrant nocturnal cortisol has been shown in women with advanced breast cancer (Zeitzer et al., 2016). Elevated cortisol levels have been observed in patients with high tumor grade and advanced-stage cancer (Rasmuson et al., 2001; Bernabé et al., 2012) as well as in those undergoing chemotherapy or target therapy (Brassard et al., 2011; Ramírez-Expósito et al., 2021). However, Colombo et al. (2019) have recently evaluated basal and stimulated adrenal function in 12 thyroid cancer patients receiving tyrosine kinase inhibitors and demonstrated a progressive ACTH increase with normal plasma cortisol levels in 10 patients, whereas the diagnosis of primary adrenal insufficiency was confirmed in 6 out of 10 patients after the detection of a blunted plasma cortisol response upon ACTH stimulation. Dysregulation of HPA axis and associated cortisol secretion can lead to immunosuppression (Sephton et al., 2009), as well as to an increased risk for anxiety and depression in cancer patients (Weinrib et al., 2010). Hence, both cancer and its treatments can alter cortisol levels and stress responses, negatively influencing quality of life and clinical outcomes in these patients (Cruz et al., 2022).

In summary, the influence of chronic stress on cancer has been extensively studied and conflicting results demonstrating harmful effects, no association and even some protective effects have been reported (Singh et al., 2021). However, considerably less attention has been paid to the inverse direction of stress/cancer relationship (cancer-to-stress relation). Further studies focusing on the reciprocal relationship between stress and cancer are needed to clarify a complex nature of this crosstalk as well as to identify common underlying mechanisms and potential susceptibility/risk biomarkers predicting worse clinical outcomes of cancer patients.

## Low-grade inflammation as a common feature underlying the crosstalk between stress and cancer

**FIGURE 2:**
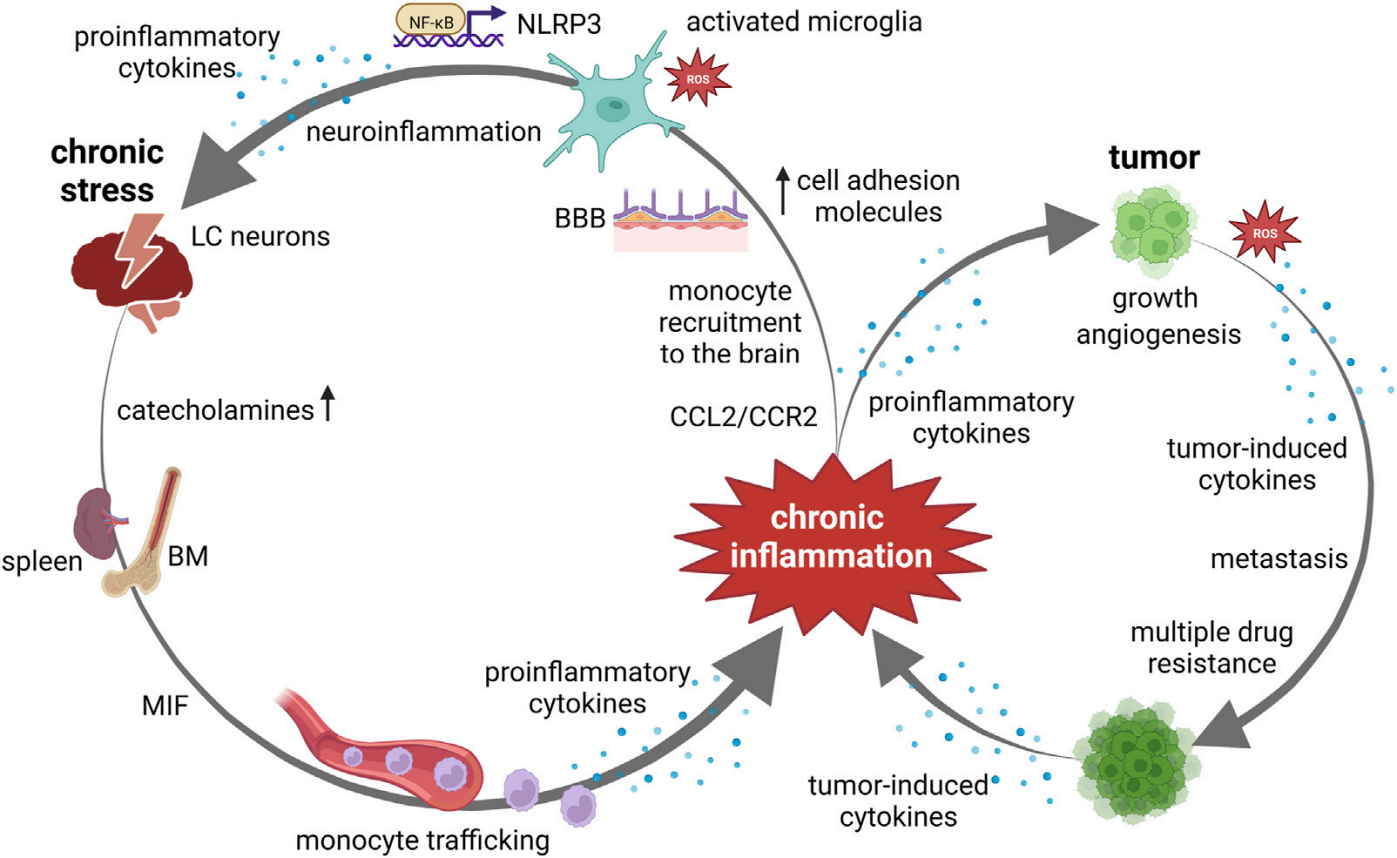
Chronic inflammation at the crossroads between chronic stress and cancer. Chronic stress, *via* catecholamine release, induces monocyte recruitment from bone marrow (BM) and spleen to the brain. Monocyte trafficking is induced *via* upregulated macrophage migration inhibitory factor (MIF) and C–C ligand 2—(CCL2)/C–C chemokine receptor 2 (CCR2) pathway. An increased expression of cell adhesion molecules in the cerebral endothelium facilitates the adherence and extravasation of peripherally derived monocytes, through blood brain barrier (BBB). Chronic stress activates microglia by altering the cerebral microenvironment through the production of proinflammatory cytokines and reactive oxygen species (ROS). Increase in ROS production triggers NF-κB-mediated NOD-like receptor protein 3 (NLRP3) inflammasome activation and subsequent proinflammatory cytokine secretion. Activation of this pathway in hippocampal microglia mediate chronic stress-induced neuroinflammation. Neuroinflammation may cause alterations in locus coeruleus (LC)-norepinephrine function resulting in overeactivity to subsequent stressors. Chronic inflammation contributes to tumor growth, angiogenesis and metastasis. Cancers with high expression levels of proinflammatory cytokines exhibit multiple drug resistance. Tumor-induced ROS and proinflammatory cytokine release further activates proinflammatory/oncogenic signaling pathways, creating the positive feedback loops.

An increased risk of cancer incidence and mortality has been linked to the systemic low-grade inflammation in numerous studies (Nøst et al., 2021). Persistent low-grade inflammation is a common pathophysiological mechanism underlying various chronic conditions including stress and cancer. Chronic psychological stress induces a sustained and marked increase in circulating pro-inflammatory factors (Miller et al., 2019), thus leading to a low-grade inflammation in peripheral tissues and brain (Rohleder, 2014). Chronic inflammation and cancer are mutually intertwined conditions, both driven by activation of common signaling pathways including signaling *via* nuclear factor kappa B (NF-kB), signal transducer and activator of transcription 3 (STAT3), and mammalian target of the rapamycin (mTOR). These pathways orchestrate the production of proinflammatory cytokines, which in turn regulate their activation (D'Orazi et al., 2021). Hence, synergistic activation of NF-kB and STAT3 induces high levels of *FAT10* gene expression and consequently counteracts the activity of tumor suppressor p53 (Choi et al., 2014). The inactivation of the tumor suppressor p53 is usually a result of *TP53* gene mutation and occurs in most human cancers. Romeo et al. (2021) have shown that mutant form of p53 protein facilitates cancer cell survival by an enhanced production of intracellular reactive oxygen species (ROS), secretion of proinflammatory cytokines, activation of mTOR signaling as well as decreased autophagic activity and uncoupling protein 2 (UCP2) expression. Moreover, these authors have pointed towards the restricting effect of wild type p53 protein on cancer cell survival induced by *TP53* gene mutation. On the other hand, this proinflammatory cytokine release further activates proinflammatory/oncogenic signaling pathways, creating the positive feedback loops, and thereby promoting a tumor-supportive milieu (D'Orazi et al., 2021). In addition to inflammation-supported oncogenic transformation, an enhanced proinflammatory cytokine release can also be triggered by stress- or cancer-associated hypoxic microenvironment, metabolic alterations as well as by anticancer therapy (Kartikasari et al., 2021). The proinflammatory cytokines contribute to tumor growth and metastasis by regulating cancer cell proliferation and invasiveness, inducing epithelial–mesenchymal transition, or by controlling tumor angiogenesis *via* upregulation of the expression of vascular endothelial growth factor and its receptors (Yadav et al., 2011; Chung et al., 2017; Kartikasari et al., 2021). Furthermore, cancer cells with high expression levels of proinflammatory cytokines exhibit multiple drug resistance, most likely through an autocrine forward-feedback loop (Kartikasari et al., 2021). Thus, a systemic proinflammatory state induced by either chronic stress or cancer is persistently maintained *via* positive feedback loops and, apart from further influencing cancer biology, may also lead to neuroinflammation (Figure 2).

The neuroinflammation caused by systemic proinflammatory state occurs through multiple mechanisms including monocyte trafficking, microglial activation, and blood–brain barrier disruption (Wohleb et al., 2015; Sun et al., 2022). Studies using rodent models have demonstrated that chronic stress induces the recruitment of monocytes from the bone marrow and spleen to the brain *via* persistent catecholamines release (Weber et al., 2017). Peripherally derived monocytes than shift their phenotypic and functional characteristic towards a hyperinflammatory state (Barrett et al., 2021) and enhance their trafficking to the brain (Wohleb et al., 2015). An increased expression of cell adhesion molecules in the cerebral endothelium under chronic inflammatory state facilitates the adherence and extravasation of peripherally derived monocytes, which subsequently differentiate into microglia-like cells. Interestingly, emerging evidence suggests that this chronic stress-induced monocyte migration, which is strongly dependent on C–C chemokine receptor 2 (CCR2) activation, however, is not associated with blood–brain barrier dysfunction (Hu et al., 2022). Furthermore, macrophage migration inhibitory factor (MIF), a proinflammatory cytokine that is markedly upregulated under chronic stress (Vignjević Petrinović et al., 2016) and induces monocyte recruitment *via* C–C ligand 2—(CCL2)/CCR2 pathway (Gregory et al., 2006), has been recognized as a key player in chronic neuroinflammation (Nasiri et al., 2020). During neuroinflammation, MIF has been identified as an upstream regulator of IL-1β and IL-6 production from microglia. Chronic stress activates microglia in multiple brain regions, thereby altering the cerebral microenvironment through the production of proinflammatory cytokines, the induction of ROS, and phagocytosis (Schramm and Waisman, 2022). An excessive production of ROS by microglia can create a self-reinforcing cycle of microglial activation and potentiate HPA axis stimulation through the release of IL-1β within the hypothalamus (Ramirez et al., 2017). In particular, glucocorticoid-induced increase in ROS production triggers NF-κB-mediated NOD-like receptor protein 3 (NLRP3) inflammasome activation and subsequent IL-1β secretion (Feng et al., 2019), and the activation of this pathway in hippocampal microglia has been suggested to mediate chronic stress-induced neuroinflammation. Most importantly, McCaffrey et al. (2022) have revealed that cancer and chronic stress activates microglia to the same extent in the same brain regions. Therefore, both chronic stress- and cancer-activated microglia can contribute to the dysfunctional neuroendocrine-immune response by reinforcing stress-related neurocircuitry.

## Therapeutic potential of targeting inflammation in stress-cancer interrelationship

The low-grade inflammation that occurs as a result of chronic stress- and/or cancer-induced dysregulation of the neuroendocrine-immune interactions is constantly maintained *via* positive feedback loops. Disrupting these positive feedback mechanisms may represent a promising therapeutic approach for cancer treatment. Over the past decade, the results derived from preclinical studies have been successfully translated into cancer immunotherapies, but therapeutic resistance develops over time in the vast majority of patients (Hou et al., 2021). Importantly, the resistance to therapy is often attributable to a proinflammatory state in cancer patients.

Cytokine secretion is commonly dysregulated during chronic stress and growing evidence points toward dysregulated cytokine release as a key feature of cellular mechanisms underlying the tumor invasiveness and drug-resistance (Kartikasari et al., 2021). Since IL-1β is one of the crucial stress-induced cytokines that promote cancer growth and metastasis, its blockers are currently being evaluated in clinical trials for cancer therapy. A recent clinical trial tested the use of anti-interleukin-1β therapy in cardiovascular disease and unexpectedly revealed that IL-1β blockade led to a significantly lower incidence of lung cancer (Ridker et al., 2017). However, sepsis occurs far more often in patients treated with IL-1β blockade than with placebo, indicating a need for further clinical research. In addition, the secretion of IL-1β is tightly regulated by NLRP3 inflammasome and targeting the inflammasome has emerged as a new therapeutic strategy for cancer (Missiroli et al., 2021). Moreover, MIF is needed for NLRP3 inflammasome activation and inhibition of MIF in macrophages results in blockade of NLRP3 inflammasome-mediated cytokine release (Wen et al., 2021). Considering an important role of MIF in NLRP3 activation in human peripheral blood monocytes, MIF inhibition may prevent stress-induced monocyte migration and consequent neuroinflammation or may be considered as a potential strategy to suppress tumor growth and overcome therapeutic resistance in some cancer patients (Samadi et al., 2015; de Azevedo et al., 2020). A distinctive “transcriptional fingerprint” of human peripheral monocytes under chronic stress conditions, characterized by increased proinflammatory gene expression, may serve as a predictive biomarker of dysregulated immune cytokine signaling in cancer patients (Kartikasari et al., 2021). Likewise, IL-6 signaling is one of the most frequently dysregulated pathways in both chronic stress and cancer. However, the results form clinical trials have demonstrated a poor clinical response to IL-6 signaling blockade in patients with multiple myeloma, breast, lung, or prostate cancers (Hou et al., 2021). Therefore, although the result from experimental studies strongly suggests that anti-inflammatory therapy has a great potential in combating cancer as well as in improving the efficacy of current treatment modalities, clinical trials to confirm the therapeutic potential of targeting inflammation in cancer treatment or prevention are still ongoing.

## Conclusion

Chronic stress and cancer are mutually interconnected conditions that can affect each other *via* bidirectional communication. In contrast to numerous research results emphasizing the influence of chronic stress on tumor initiation, growth and metastasis, the reciprocal effects of tumor on stress responses have been far less studied. The systemic low-grade inflammation, which can be induced by both chronic stress and cancer, has emerged as a significant contributor to tumor invasiveness and cancer treatment resistance. Therefore, the anti-inflammatory approaches have a huge therapeutic potential for cancer treatment, but the translation of experimental results into clinically effective anti-cancer therapy that targets inflammation is still pending. Further studies focusing on reciprocal causal relationship between chronic stress and cancer are needed to unravel the complex dynamics of underlying neuroendocrine-immune crosstalk. To elucidate the mechanisms underlying this complex crosstalk, the integrative multi-omics approaches (e.g., genomics, metabolomics, proteomics) should be considered. Better understanding how cancer alters stress responsivity may provide novel insights into mechanisms contributing tumor immune evasion and therapy resistance, as well as to point towards the susceptibility/risk biomarkers predicting worse clinical outcomes in cancer patients. Furthermore, multi-omics analysis of peripheral blood cells may enable the identification of distinct cytokine signatures as potential biomarkers assessing the risk for stress-related cancer development and/or progression.
